# Cardiovascular Toxicity Associated With Immune Checkpoint Inhibitors: Interpreting the Discrepancy Between Clinical Trials and Real-World Data

**DOI:** 10.7759/cureus.87049

**Published:** 2025-06-30

**Authors:** Amalia Papanikolopoulou, Helena Michalopoulou, Konstantinos G Kyriakoulis, Polyxeni Alexiou, Nikolaos Syrigos, John Vathiotis, Costas Thomopoulos, Andriani Charpidou

**Affiliations:** 1 Third Department of Internal Medicine, Sotiria General Hospital, School of Medicine, National and Kapodistrian University of Athens, Athens, GRC; 2 Department of Oncology, Hippokration General Hospital, Athens, GRC; 3 Department of Cardiology, Laiko General Hospital, Athens, GRC

**Keywords:** cancer, cardiovascular toxicity, immune checkpoint inhibitors, myocarditis, observational studies

## Abstract

Real-world data on cardiovascular immune-related adverse events (CV-irAEs) in cancer patients treated with immune checkpoint inhibitors (ICIs) present findings that differ from those reported in meta-analyses of clinical trials. This study aims to estimate the incidence of CV-irAEs from observational studies among patients undergoing ICI therapy for various malignancies and investigate the discrepancy between the results of meta-analyses and observational studies. A systematic literature review and meta-analysis were conducted according to the Preferred Reporting Items for Systematic reviews and Meta-analyses (PRISMA) guidelines. The PubMed database was searched for observational studies that included cancer patients treated with ICIs. Α single-arm meta-analysis using the *metaprop* command in Stata Statistical Software: Release 16 (StataCorp LLC, College Station, Texas, United States) was performed for the following outcomes: myocarditis, pericardial disease, arrhythmias, cardiac failure, Takotsubo cardiomyopathy, ischemic heart disease, heart valve disease, venous thromboembolism and artery disease. ICI treatment agents were classified into three major classes: PD-1 inhibitors, PD-L1 inhibitors, CTLA4 inhibitors, or their combinations. Heterogeneity was quantified using the I2 statistic and small study effect, and potential publication bias was assessed by inspecting funnel plots, as well as by Egger’s test. A total of 42 studies were included. The incidence of CV-irAEs within the entire population undergoing treatment with ICIs was assessed as follows: total CV-irAEs: 8% (95% confidence interval (CI): 6%, 10%), arrhythmias: 18% (95%CI: 10%, 27%), myocarditis: 11% (95%CI: 5%, 18%), cardiac failure: 8% (95%CI: 2%, 15%), ischemic heart disease: 6% (95%CI: 3%, 11%), pericardial disease: 5% (95%CI: 1%, 10%), artery disease 5% (95%CI: 1%, 12%), and venous thromboembolism: 3% (95%CI: 0%, 8%); cardiomyopathy and heart valve disease had minimal number of observed episodes, thus the pooled incidence results are referring as zero, 0% (95%CI: 0%, 0%) and total CV deaths: 1% (95%CI: 0%, 3%). Median time to CV-irAEs was estimated at 119 days (interquartile range (IQR) 53-180). The most common CV-irAEs were arrhythmias, myocarditis, and cardiac failure with life-threatening complications. Data derived from meta-analyses of clinical trials in most cases indicated that the total incidence of CV-irAEs varied between 0.05% and 1.30%, while in large pharmacovigilance databases, it ranged from 0.125% to 6.7%. In our meta-analysis of post-market surveillance studies, higher estimates were obtained, which offer an insight into the long-term prevalence and outcomes for patients experiencing CV complications associated with ICIs. Longer follow-up period and different definitions of cardiotoxicity may account for the higher cardiotoxicity rates that seem to reflect an emerging threat.

## Introduction and background

Over the last decade, immune checkpoint inhibitors (ICIs) have changed the treatment of cancer, with indications that continue to expand and largely cover the entire spectrum from palliative to the curative setting across many tumor types [1]. ICIs are monoclonal antibodies that block down-regulators of adaptive immunity, including cytotoxic T lymphocyte-associated protein 4 (CTLA-4), programmed cell death protein 1 (PD-1), and its ligand (PD-L1), unleashing effector T-cell activity and reinstating anti-tumor activity [2]. Disproportionate stimulation of the immune system may be the cause of a wide range of inflammatory side effects called immune-related adverse events (irAEs) that might essentially affect every organ system [2]. Cardiovascular (CV) irAEs arise in response to the accumulation of activated T-cells in the cardiac tissues, within the myocardium, pericardium, vasculature, and conduction system, which is followed by neutrophil and macrophage infiltration [3]. This pathophysiological diversity has led to emerging clinical syndromes in cardio-oncology not previously defined [4].

According to phase III clinical trials of combined nivolumab (anti-PD1) and ipilimumab (anti-CTLA-4) or monotherapy in untreated melanoma [5,6], CV-irAEs were infrequent, although associated with some irreversible, life-threatening, and fatal complications [7]. The limited sample size and shorter follow-up, along with the absence of a precise definition and the underlying various mechanisms of cardiotoxicity, suggest that CV-irAEs might be under-reported in immunotherapy trials [4]. Along with the expanding use of ICI therapy, an increasing number of cases and cohort reports have described a variety of clinical presentations and a spectrum of CV-irAEs with mild signs and symptoms to fulminant and potentially fatal events [8]. Emerging real-world data from pharmacovigilance and observational studies [9,10] provided estimations for the incidence, outcomes, and predictors of these rare events, highlighting a discrepancy between the findings of clinical trials and real-world data. This ascertainment has contributed to the development of systematic diagnostic criteria to enhance case identification and reporting [4].

From professional organizations, the first structured approach to the definitions, diagnosis, treatment, and prevention of cancer therapy-related CV toxicity came from the European Society of Cardiology (ESC) in 2022 [11]. Consensus definitions were used for cardiomyopathy and heart failure, myocarditis, vascular toxicities, hypertension, cardiac arrhythmias, while for pericardial and valvular heart diseases, the definitions were used as for the general cardiology population. Most importantly, the ESC cardio-oncology guidelines acknowledged the need for multidisciplinary collaboration and further research due to current evidence limitations. Furthermore, numerous meta-analyses of RCTs have been conducted in an effort to evaluate more representative outcomes concerning the incidence of CV-irAEs [12-15]. Conversely, the synthesis of data from cohort studies was not utilized due to the possible heterogeneity in definitions, populations, or outcomes observed in real-world settings.

The aim of this review and meta-analysis was to estimate the pooled incidences of CV-irAEs derived from observational studies carried out from the post-authorization phase of ICIs until March 2024. Under the term of ICI-related CV toxicities, we have included myocarditis, pericardial disease, arrhythmias, cardiac failure, Takotsubo cardiomyopathy, ischemic heart disease, heart valve disease, venous thromboembolism, and artery disease [16].

## Review

Methodology

Design

All authors collectively reached a consensus on the study design prior to its implementation. We employed a PICO-S (Population, Intervention, Comparison, Outcomes, Study selection) strategy tool to enhance our search strategy [17]. The population included cancer patients, while the intervention was treatment with ICIs. A comparison group was not a prerequisite. The outcome of interest was CV toxicity, and we performed a search to identify any type of relevant study. The PICO-S approach is demonstrated in Table 1.

**Table 1:** PICO-S approach for search strategy PICO-S: Population, Intervention, Comparison, Outcomes, Study selection

Particulars	Inclusions
Population	Cancer patients
Intervention	Immune checkpoint inhibitors
Comparison	Optional
Outcome	Cardiovascular toxicity
Study selection	Observetional; prospective; retrospective studies

Reporting and Protocol Registration

**Figure 1:**
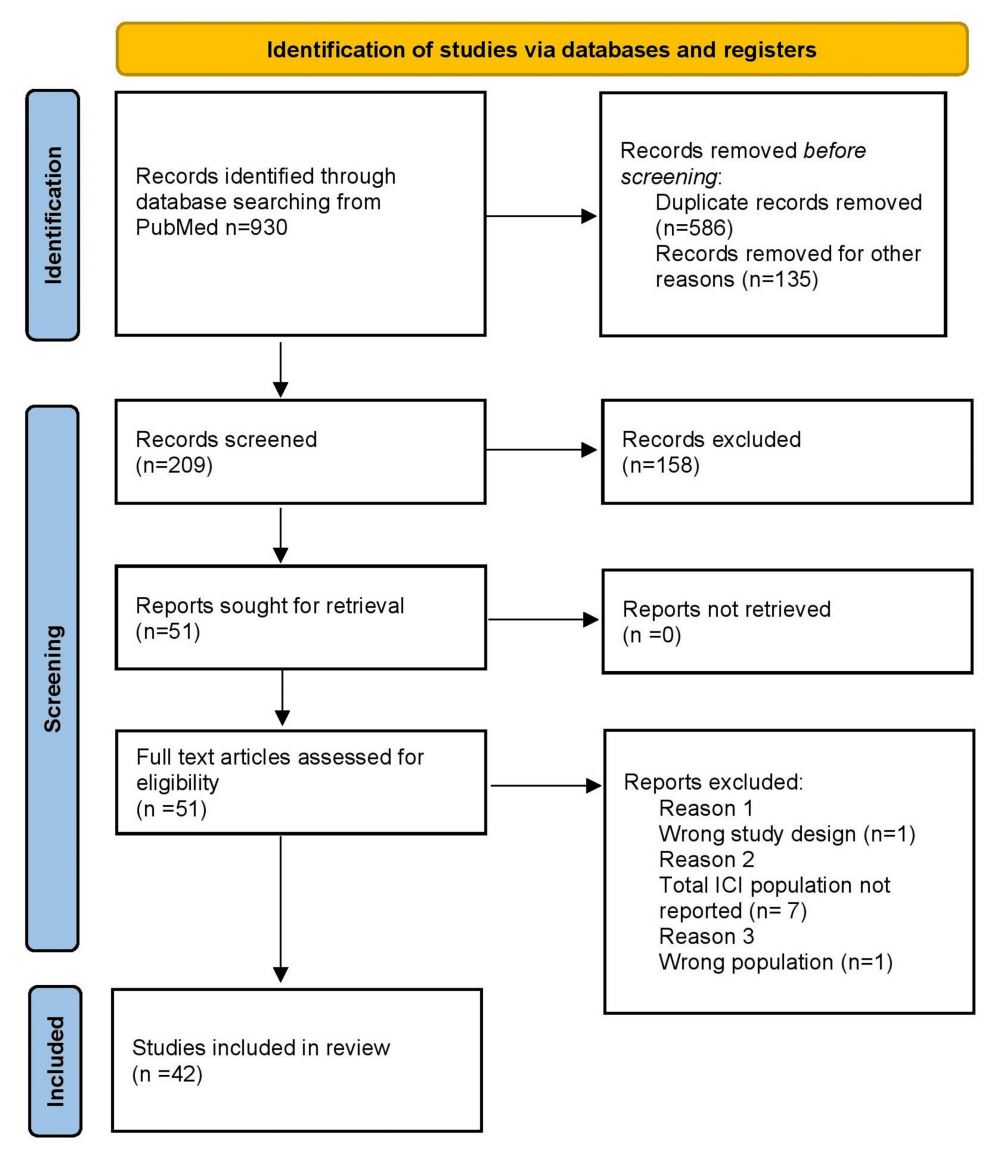
PRISMA 2020 flow diagram for systematic reviews and meta-analyses of studies selection For more information, visit: http://www.prisma-statement.org/ [17]

This study was conducted as a systematic review in accordance with the Preferred Reporting Items for Systematic Reviews and Meta-Analyses (PRISMA) guidelines [18]. The PRISMA 2020 flow diagram for systematic reviews and meta-analyses is presented in Figure 1. The protocol was registered in the PROSPERO international prospective register of systematic reviews (CRD42024592961).

Search Strategy

A systematic search of PubMed was performed until March 22, 2024 using the following search algorithm: (“immune checkpoint inhibitors cardiotoxicity” OR “immune checkpoint inhibitors cardiovascular toxicities” OR “immune checkpoint inhibitors cardiac adverse events”) AND (“anti-PD-1” OR “PD-1” OR “programmed death ligand-1” OR “PD-L1” OR “anti-PD-L1” OR “programmed death ligand-1” OR “CTLA-4” OR “anti-CTLA-4” OR “cytotoxic T-lymphocyte antigen-4” OR “pembrolizumab” OR “nivolumab” OR “ipilimumab” OR “durvalumab” OR “avelumab” OR “cemiplimab” OR “atezolizumab”).

Inclusion and Exclusion Criteria

Studies were selected and screened based on title and abstract using the inclusion/exclusion criteria. After screening, all selected full-text articles were assessed for eligibility. We included prospective and retrospective cohort studies, both matched and unmatched, providing categorical data on the outcomes of interest. The criteria for inclusion encompassed: (a) malignancies from solid organs except gynecological cancers, (b) ICI treatment agents were referring to three major classes: PD-1 inhibitors, PD-L1 inhibitors, CTLA4- inhibitors, or their combinations, and (c) any CV toxicity classified into nine major categories: myocarditis, pericardial disease, arrhythmias, cardiac failure, Takotsubo cardiomyopathy, ischemic heart disease, heart valve disease, venous thromboembolism, artery disease [16].

Accordingly, we excluded studies with no exposure of the total population. We have also omitted non-human studies, letters to the editor (not containing primary data), case reports, editorial articles, commentaries, reviews, meta-analyses, pre-authorization clinical trials, pharmacovigilance studies, and articles with no authors listed. Other restriction criteria were hematological malignancies, combined irAEs, a combination of ICIs with TKIs or anti-VEGF therapies, or anthracyclines. Studies were limited to the English language since study resources precluded any translation activities. Unpublished data, such as abstracts and posters, were excluded. Publications not providing a full text and reports that were not peer-reviewed were excluded.

Data Extraction and Tabulation

The literature search, selection of studies, and extraction of data were performed by three independent reviewers (AP, JV, HM), and disagreements were resolved by discussion with senior authors. For each study, we extracted the data concerning the primary characteristics of patients (age, gender, tumor type, type of immunotherapeutic agent, concurrent/previous systemic therapies, comorbidities, concurrent CV complications, time to CV-irAEs presentation, outcome). The history of CV disease (CVD) and risk factors for irAEs were recorded. The outcome of interest was the incidence of CV-irAEs in the study population. Consequently, information regarding the effects of CV-irAEs on the overall management of cancer, as well as various baseline clinical characteristics (such as Eastern Cooperative Oncology Group (ECOG) Performance Status and non-CV-irAEs), was omitted from the descriptive analysis. The remaining clinicopathological characteristics were compiled based on the evidence available from published literature.

Definitions

The consensus statement from the International Cardio-Oncology Society (IC-OS) provided a structure for defining CV toxicities of cancer therapy commonly reported in clinical studies [16]. Recent studies have broadened the understanding of the impact of ICI therapy on the CV system, revealing additional effects such as an increase in cardiac dysfunction without myocarditis, arrhythmias, venous thromboembolic disease, accelerated atherosclerosis, and atherosclerosis-related CV events [19].

In our study, the term pericardial disease refers to pericarditis and pericardial effusion. The category of arrhythmias encompasses atrial fibrillation (AF), conduction abnormalities, heart block, cardiac arrest, cardiac death, cardiogenic shock, and syncope. Ischemic heart disease is represented by acute coronary syndrome (ACS), myocardial infarction (MI), ST-elevation MI (STEMI), non-ST-elevation MI (NSTEMI), and unstable angina. Venous thromboembolism includes deep vein thrombosis (DVT) and pulmonary embolism (PE). Artery disease encompasses clinical conditions such as stroke, transient ischemic attack (TIA), carotid artery disease, peripheral artery disease (PAD), arterial thromboembolism (ATE), vasculitis, pulmonary artery hypertension, and acute limb ischemia.

Statistical Analysis

Meta-analysis of proportions was performed to assess the pooled incidence of CV-irAEs. The Freeman-Tukey double-arcsine-transformed proportion was computed for each study. Confidence intervals were computed using the Score (Wilson) procedure. Heterogeneity was quantified using the I² statistic, and a random-effects model was used (DerSimonian-Laird approach) when the I² statistic value was ≥50%. Small study effect and potential publication bias were assessed by inspecting funnel plots (proportions plotted against respective standard errors), as well as by Egger’s test [20]. Respective meta-analyses were performed to compute the pooled measures of baseline characteristics of included studies. Results were expressed as mean (95% confidence intervals (CI) for continuous variables and as percentage (95%CI) for categorical variables. Analyses were performed mainly using the metaprop command in Stata Statistical Software: Release 16 (StataCorp LLC, College Station, Texas, United States). Two-sided P <0.05 was considered statistically significant.

Results

Literature Search and Study Inclusion

Of 930 articles initially retrieved, 42 fulfilled the inclusion criteria and were finally included in the systematic review and meta-analysis [21-62]. The pooled baseline characteristics of the included studies are shown in Table 2. A total of 54,409 subjects treated with ICIs were included, of whom 4579 (8%, 95%CI: 6%, 10%) suffered at least one CV-irAE. The average age of participants was 68.2 years, and nearly one-third were female. Most of the participants received PD-1 inhibitors (81%). The most prevalent CV comorbidities were hypertension (36%), diabetes (14%), and dyslipidemia (6%). The most common type of cancer was lung cancer (59%), followed by melanoma (11%). A more detailed summary of baseline characteristics of each of the included studies can be found in Table 3.

**Table 2:** Pooled baseline characteristics of participants of the included studies CTLA-4: cytotoxic T-lymphocyte-associated antigen 4; CV-irAEs: cardiovascular immune-related adverse events; ICIs: immune checkpoint inhibitors; N: number; PD-1: programmed death 1; PD-L1: programmed death-ligand 1 All proportions except “Number of irAEs” are computed using the "Number of irAEs” as the denominator Data given as mean or percentage (95%CI), unless otherwise indicated

Pooled Baseline Characteristics	Mean or % (95% Confidence Intervals)
Number of patients treated with ICIs	54409
Number of CV-irAEs	4579
CV-irAEs, % (95%CI)	8 (6, 10)
Females, % (95%CI)	33 (29, 36)
Age (years), mean (95%CI)	68.2 (66.8, 69.8)
ICIs received, % (95%CI)	
PD-1 inhibitors	81 (73, 87)
PD-L1 inhibitors	5 (3, 8)
CTLA-4 inhibitors	2 (0, 7)
ICIs combination	2 (0, 4)
Comorbidities, % (95%CI)	
Ischemic Heart disease	6 (3, 9)
Cardiac Failure	2 (0, 4)
Hypertension	36 (22, 50)
Diabetes Mellitus	14 (9, 20)
Dyslipidemia	6 (2, 12)
Arrhythmias	0 (0, 1)
Heart Valve Disease	0 (0, 0)
Cerebrovascular Disease	0 (0, 2)
Venous Thromboembolism	0 (0, 0)
Peripheral Vascular Disease	0 (0, 1)
Cancer type, % (95%CI)	
Lung	59 (47, 70)
Melanoma	11 (5, 20)
Genitourinary	1 (0, 3)
Gastrointestinal	3 (0, 7)
Other	4 (0, 11)

**Table 3:** Baseline characteristics of participants of the included studies NR: not reported

Study (Authors, year)	Total Cases	Number of irAEs	Female Patients, %	Age (years)	PD-1 inhibitors, %	Hypertension, %	Diabetes, %	Lung cancer, %	Melanoma, %
Ho et al., 2023 [21]	407	18	NR	NR	100	NR	NR	NR	NR
Son et al., 2023 [22]	194	22	NR	NR	NR	NR	NR	100	0
Tan et al., 2023 [23]	366	26	30.8	Median 68 (IQR 61–72)	69.2	61.5	15.4	73.1	11.5
Toribio-García et al., 2023 [24]	195	3	NR	Median 76 (IQR 70–88)	NR	0	0	NR	NR
Xiao et al., 2023 [25]	487	12	33.3	Median 67.5 (IQR 58.3–71.8)	100	25	50	25	0
Reyes-Gibby et al., 2023 [26]	453	143	NR	NR	NR	0	0	0	0
He et al., 2023 [27]	673	14	28.6	Mean 60.43 (range 46.33-74.53)	100	21.4	14.3	100	0
Li et al., 2022 [28]	5518	691	36.8	NR	41.4	50.8	17.4	42.3	32.7
Brumberger et al., 2022 [29]	538	34	61.5	Mean 65 (range 57-73)	94.1	53.8	7.7	88.2	0
Li et al., 2022 [30]	291	9	11.1	Mean 64 (range 44-66)	88.9	44.4	22.2	0	0
Chen et al., 2022 [31]	1047	73	23.2	Mean 59.8 (range 48.1-71.5)	89	23.3	19.2	42.5	0
Torrente et al., 2022 [32]	378	65	38.5	Mean 64.82 (range 52.5-67.1)	NR	44.6	15.4	NR	NR
Isawa et al., 2022 [33]	129	35	20	Median 71 (IQR 65-78)	85.7	51.4	14.3	100	0
Wu et al., 2022 [34]	580	23	13	Mean 72 (range 65.2-78.8)	87	47.8	69.6	47.8	13
Laenens et al., 2022 [35]	672	69	NR	NR	NR	NR	NR	NR	NR
Wu et al., 2022 [36]	495	64	18.8	Mean 61.78 (range 51.81-71.75)	87.5	NR	NR	57.8	0
Andres et al., 2022 [37]	2647	89	44.9	Median 63 (IQR 51-72)	49.4	28.1	6.7	NR	NR
Wang et al., 2022 [38]	289	22	NR	Median 78.7 (IQR NA)	66.7	NR	NR	0	100
Kurozumi et al., 2022 [39]	32	9	NR	NR	NR	NR	NR	100	NR
Chiang et al., 2022 [40]	868	74	NR	NR	64.9	NR	NR	NR	NR
Faubry et al., 2022 [41]	99	3	NR	NR	33.3	0	0	100	0
Chan et al., 2022 [42]	713	24	NR	NR	100	NR	NR	100	0
Zhou et al., 2022 [43]	1959	320	31.6	Mean 64 (range 51.2-76.8)	96.6	15.3	9.4	NR	NR
Joseph et al., 2021 [44]	268	4	0	Mean 71.5 (range 65-78)	0	75	50	0	100
Waheed et al., 2021 [45]	424	62	37.1	Mean 64.3 (range 54.1-74.5)	71	58.1	22.6	32.3	25.8
Jain et al., 2021 [46]	12187	1541	37.4	NR	62.9	NR	30.5	42.1	38.9
Puzanov et al., 2021 [47]	1001	23	39.1	Median 73.25 (IQR 64.4-79.5)	91.3	82.6	13	13	56.5
Heilbroner et al., 2021 [48]	4960	418	28.2	Mean 65.6 (range 54.3-76.9)	68.7	0	12.7	63.6	5.5
Gong et al., 2021 [49]	2842	42	52.3	ΝR	78.6	52.4	9.5	64.3	19
Sussman et al., 2021 [50]	228	51	ΝR	ΝR	43.1	NR	NR	0	92.2
Moik et al., 2021 [51]	672	56	NR	NR	80.4	NR	NR	25	32.1
Deschênes-Simard et al., 2021 [52]	593	65	41.5	Median 63.8 (IQR 57-69.7)	83.1	29.2	0	90.8	0
Moey et al., 2020 [53]	196	23	34.8	Mean 68.7 (range 66.9-70.5)	100	52.2	30.4	100	0
Chahine et al., 2020 [54]	2830	9	44.4	Mean 70 (range 61-79)	66.7	77.8	11.1	55.6	22.2
D’Souza et al., 2020 [55]	1100	87	NR	NR	83.9	NR	NR	75.9	24.1
Canale et al., 2020 [56]	60	4	NR	NR	100	NR	NR	100	0
Oren et al., 2020 [57]	3326	80	32.5	Mean 65.4 (range NR)	NR	30	27.5	25	17.5
Wang et al., 2020 [58]	283	3	100	Mean 69.6 (range NR)	100	66.7	0	0	33.3
Drobni et al., 2020 [59]	2842	139	NR	NR	NR	NR	NR	NR	NR
Nichetti et al., 2020 [60]	217	30	30	Median 70 (IQR 56-83)	60	20	10	100	0
Chitturi et al., 2019 [61]	135	69	NR	NR	NR	NR	NR	100	0
Bar et al., 2019 [62]	1215	31	22.6	Mean 66.61 (range NR)	83.9	58.1	25.8	41.9	35.5

Synthesis of Included Studies

**Figure 2:**
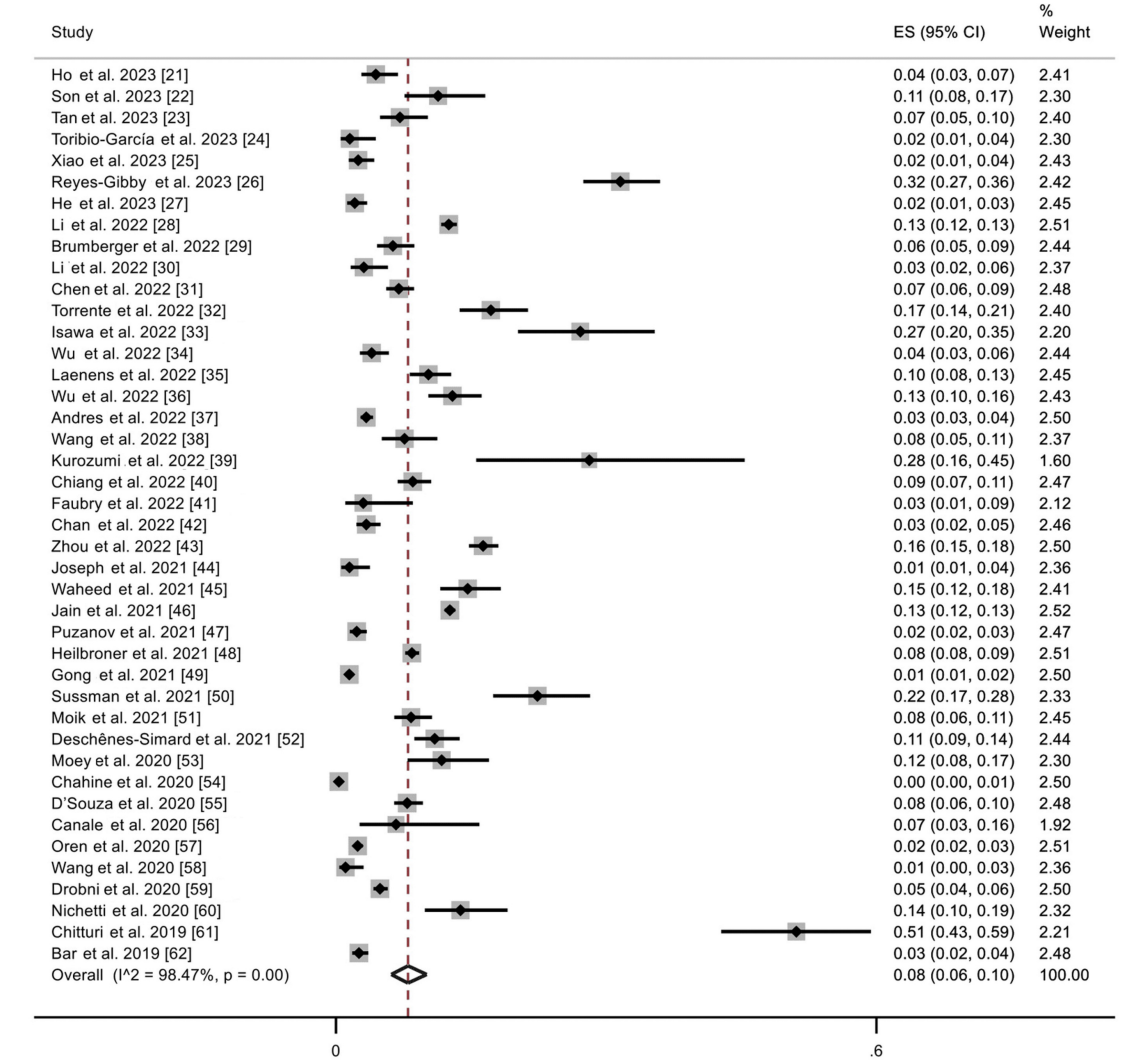
Meta-analysis of proportions of 42 included studies showing the incidence of CV-irAEs ES: effect size References: [21-62]

Out of the 54,409 participants, 4579 suffered an episode of CV-irAEs during a median follow-up of 119 days, resulting in a pooled incidence of CV-irAEs of 8% (95%CI: 6%, 10%) (Figure 2).

Most frequent CV-irAEs were arrhythmias (18% (95%CI: 10%, 27%)), myocarditis (11% (95%CI: 5%, 18%)), cardiac failure (8% (95%CI: 2%, 15%)), and to a lesser extent ischemic heart disease, pericardial disease, artery disease, and venous thromboembolism (Table 4).

**Table 4:** Immune-related cardiovascular adverse events (N=4579) CV-irAEs: cardiovascular immune-related adverse events; IQR: interquartile range Data given as percentage (95% CI), unless otherwise indicated

CV irAEs	Percentage (95% confidence intervals)
Number	4579
Time to CV irAEs, median (IQR)	119 (IQR 53-180)
Myocarditis	11 (5, 18)
Pericardial Disease	5 (1, 10)
Arrythmias	18 (10, 27)
Cardiac Failure	8 (2, 15)
Takotsubo Cardiomyopathy	0 (0, 0)
Ischemic Heart Disease	6 (3, 11)
Heart Valve Disease	0 (0, 0)
Venous Thromboembolism	3 (0, 8)
Artery Disease	5 (1, 12)

For Takotsubo cardiomyopathy and heart valve disease, the observed episodes were very few (65/54,409 and 21/54,409, respectively); thus, the pooled incidence results are <0.2% referred to as zero. The overall CV fatality rate was noted to be 1% (95%CI: 0%, 3%) of the total participants.

Risk of Bias, Small Study Effect, and Potential Publication Bias

The majority of the studies were deemed as having a moderate quality (Table 5). 

**Table 5:** The assessment of the risk of bias of the included studies for the present meta-analysis using a JBI checklist* Grading: ‘Yes’ was graded with 1, ‘Unclear’ with 0.5, ‘No’ with 0. Studies with ≥7 ‘Yes’ were categorized as of high quality; >5-<7 ‘Yes’ as of moderate quality; <5 as of low quality * JBI Checklist for Analytical Cross Sectional Studies: Critical Appraisal tools for use in JBI Systematic Reviews [63]

Study (Authors, Year)	1. Was the sample frame appropriate to address the target population?	2. Were study participants recruited in an appropriate way?	3. Was the sample size adequate?	4. Were the study subjects and the setting described in detail?	5. Was the data analysis conducted with sufficient coverage of the identified sample?	6. Were valid methods used for the identification of the condition?	7. Was the condition measured in a standard, reliable way for all participants?	8. Was there appropriate statistical analysis?	9. Was the response rate adequate, and if not, was the low response rate managed appropriately?	Quality score
Ho et al., 2023 [21]	yes	yes	no	no	yes	yes	no	yes	NA	5
Son et al., 2023 [22]	yes	yes	no	no	yes	yes	no	yes	NA	5
Tan et al., 2023 [23]	yes	yes	no	yes	yes	unclear	no	yes	NA	5.5
Toribio-García et al., 2023 [24]	yes	yes	no	yes	yes	yes	no	yes	NA	6
Xiao et al., 2023 [25]	yes	yes	no	yes	yes	yes	no	no	NA	5
Reyes-Gibby et al., 2023 [26]	yes	yes	yes	no	yes	yes	no	yes	NA	6
He et al., 2023 [27]	yes	yes	yes	yes	yes	yes	no	yes	NA	7
Li et al., 2022 [28]	yes	yes	yes	yes	yes	yes	no	yes	NA	7
Brumberger et al., 2022 [29]	yes	yes	yes	yes	yes	uclear	no	yes	NA	6.5
Li et al., 2022 [30]	yes	yes	no	yes	yes	yes	yes	unclear	NA	6.5
Chen et al., 2022 [31]	yes	yes	yes	yes	yes	yes	no	yes	NA	7
Torrente et al., 2022 [32]	yes	yes	no	yes	yes	yes	no	yes	NA	6
Isawa et al., 2022 [33]	no	yes	no	yes	yes	yes	yes	yes	NA	6
Wu et al., 2022 [34]	yes	unclear	yes	yes	yes	yes	no	yes	NA	6.5
Laenens et al., 2022 [35]	yes	yes	yes	unclear	yes	yes	no	yes	NA	6.5
Wu et al., 2022 [36]	yes	yes	no	unclear	yes	yes	no	yes	NA	5.5
Andres et al., 2022 [37]	yes	yes	yes	no	yes	yes	no	yes	NA	6
Wang et al., 2022 [38]	yes	yes	no	unclear	yes	yes	no	yes	NA	5.5
Kurozumi et al., 2022 [39]	no	yes	no	yes	yes	unclear	no	yes	NA	4.5
Chiang et al., 2022 [40]	yes	yes	yes	unclear	yes	yes	no	yes	NA	6.5
Faubry et al., 2022 [41]	yes	yes	no	no	yes	yes	yes	no	yes	6
Chan et al., 2022 [42]	yes	yes	yes	no	yes	yes	no	yes	NA	6
Zhou et al., 2022 [43]	yes	yes	yes	yes	yes	unclear	no	yes	NA	6.5
Joseph et al., 2021 [44]	yes	yes	no	yes	yes	unclear	no	yes	NA	5.5
Waheed et al., 2021 [45]	yes	yes	no	yes	yes	yes	no	yes	NA	6
Jain et al., 2021 [46]	yes	yes	yes	yes	yes	yes	no	yes	NA	7
Puzanov et al., 2021 [47]	yes	yes	yes	yes	yes	yes	no	yes	NA	7
Heilbroner et al., 2021 [48]	yes	yes	yes	yes	yes	yes	no	yes	NA	7
Gong et al., 2021 [49]	yes	yes	yes	yes	yes	yes	no	yes	NA	7
Sussman et al., 2021 [50]	yes	yes	no	no	yes	unclear	no	yes	NA	5.5
Moik et al., 2021 [51]	yes	yes	yes	unclear	yes	yes	no	yes	NA	6.5
Deschênes-Simard et al., 2021 [52]	yes	yes	yes	yes	yes	unclear	no	yes	NA	6.5
Moey et al., 2020 [53]	yes	yes	no	yes	yes	yes	no	yes	NA	6
Chahine et al., 2020 [54]	yes	yes	yes	yes	yes	yes	no	no	NA	6
D’Souza et al., 2020 [55]	yes	yes	yes	unclear	yes	yes	no	yes	NA	6.5
Canale et al., 2020 [56]	no	unclear	no	no	yes	unclear	no	no	NA	2
Oren et al., 2020 [57]	yes	yes	yes	yes	yes	unclear	no	yes	NA	6.5
Wang et al., 2020 [58]	yes	yes	no	no	yes	unclear	no	no	NA	3.5
Drobni et al., 2020 [59]	yes	yes	yes	unclear	yes	yes	no	yes	NA	6.5
Nichetti et al., 2020 [60]	yes	yes	no	yes	yes	unclear	no	yes	NA	5.5
Chitturi et al., 2019 [61]	yes	yes	no	unclear	yes	yes	no	yes	NA	5.5
Bar et al., 2019 [62]	yes	no	yes	yes	yes	unclear	no	yes	NA	5.5

**Figure 3:**
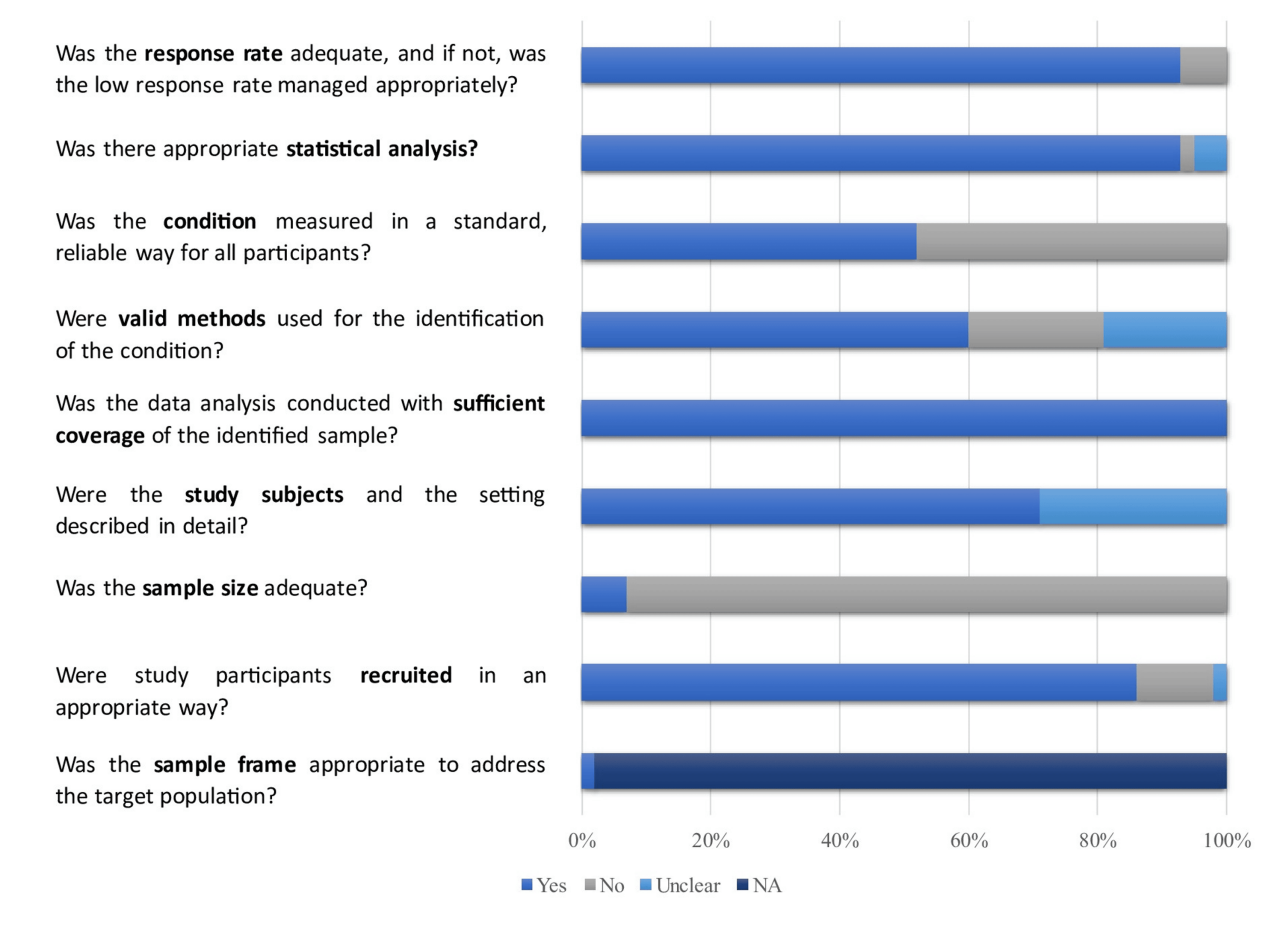
Assessment of the risk of bias of included studies

The risk of bias was assessed using the JBI Checklist for Analytical Cross Sectional Studies [63] and is presented in Figure 3.

**Figure 4:**
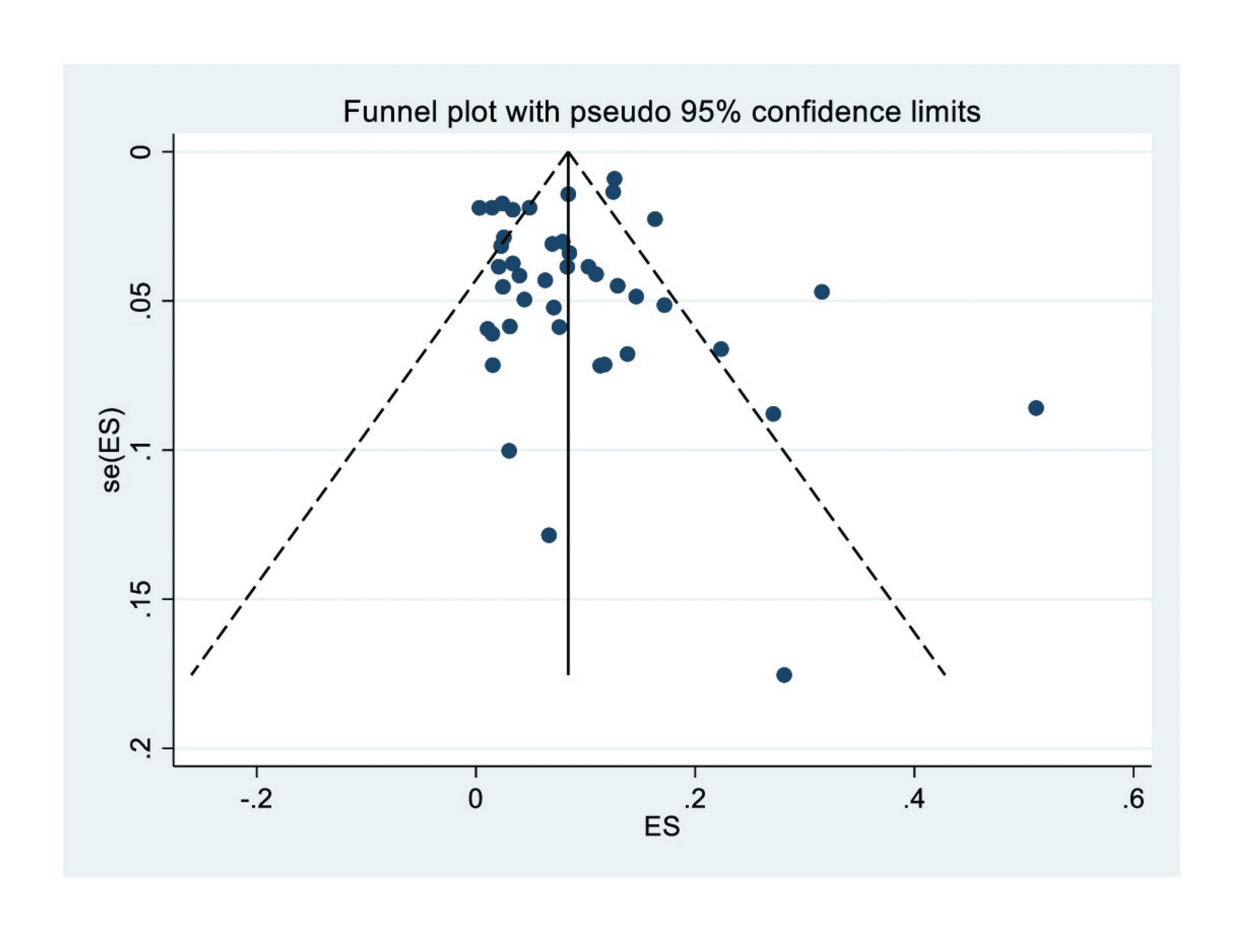
Funnel plot assessing the presence of small study effect and potential publication bias.

Egger’s test funnel plot did not reveal a small study effect (P=0.93) (Figure 4).

Cohort Studies Characteristics

The 42 observational cohort studies demonstrate a diverse range of characteristics regarding their design, the types of cancer investigated, and CV-irAEs observed within a real-world setting. Study design comprised 33 single-center studies [21-27,29,31-33,35-39,41,44,45,47,49-51,53-62], four multi-center studies [30,34,40,52], and five wider population-based studies from healthcare electronic databases [28,42,43,46,48]. The data were primarily selected retrospectively, with the exception of two studies that were designed prospectively [30,41].

Given the infrequency of these events, 26 studies encompassed patients across all types of cancer [21,23-25,28,29,31,32,34-37,40,43-49,51,54,57-59,62], whereas 11 studies specifically focused on lung cancer [22,27,33,39,41,42,52,53,56,60,61], two studies on melanoma [38,50], one study included both malignancies [55], one study enclosed patients exclusively with head and neck cancers [26], and one study with gastrointestinal cancer [30]. Furthermore, due to the rarity of these events, the investigation encompassed all categories of CV-irAEs across 21 studies [21,22,24-26,28-33,36-37,43,45,46,48,53-55,57] and the occurrence of major adverse cardiac events (MACE) in six studies, defined as the composite of CV death, cardiogenic shock, cardiac arrest, and hemodynamically significant complete heart block [34,35,38,40,42,61].

Regarding a specific CV event, four studies focused on myocarditis [27,41,47,58], four studies on thromboembolic events [50-52,60], four studies on atherosclerotic CV events [23,39,59,62], two studies on pericardial disease [49,56], and one study on arrhythmias [44]. Finally, in three observational studies, a propensity score matching between cohorts [40,43,59] was used for comparison. In three separate studies, a matched control group consisting of cancer patients was utilized; two of these studies compared this group with a population without any history of CV events [30,35], while the third study compared it to a population that had undergone chemotherapy exclusively [46].

Discussion

Epidemiological Findings of ICI CV Toxicities and Fatality Rates

CV-irAEs include a wide range of heart disorders with a complex differential diagnosis [64]. Early randomised clinical trials (RCTs) failed to reveal any association between ICIs and myocardial toxicity due to limited sample size and shorter follow-up. In phase 1 to 3 trials of ipilimumab, nivolumab, and pembrolizumab for untreated melanoma, CV toxicity was not identified as a significant irAE in the treatment group receiving the drugs [5,6].

Pooled data obtained from meta-analyses of clinical trials sourced from PubMed up to April 2022, indicate that the overall incidence of CV-irAEs varies between 0.05% and 8.32% [12,13]. Also, in large pharmacovigilance databases, the expanded use of ICI therapy has led to heightened surveillance in reportable events. ranging from 0.125% [65] to 6.7% [66]. Data from individual hospital registries using electronic healthcare records show varying overall incidences of CV-irAEs, with some reporting low rates (0.32%) [54], while others report relatively high rates of 31.5% [26]. Differences in event frequency could be explained by how the data were collected, passive vs active surveillance and heterogeneity in the definition of CV toxicities [4] Pharmacovigilance studies rely on reported events [10], whereas authors from single-center studies have the potential to conduct a thorough investigation of electronic medical records in their study population, thereby reducing the risk of underestimating CV-irAE incidence [21,29,45,54].

In our meta-analysis, the pooled incidence of all CV-irAEs was found to be 8% (95%CI: 6%, 10%) which aligns closely with the findings reported by Xavier et al. [13] but exceeding the scope of most other meta-analyses [12,14,15] and pharmacovigilance studies [65,66]. This discrepancy could be attributed to the expanded use of ICIs and heightened awareness of their potential toxicity. An alternative explanation could be the heterogeneity present among the included studies in meta-analyses, which identified considerable asymmetry and notable publication bias concerning either all or a portion of the results of the "pooled incidences" [12,14,15].

The various manifestations of cardiotoxicity in real-world settings exhibit a broad spectrum of presentations, ranging from isolated adverse events to overlapping syndromes, spanning from subclinical conditions to significant MACE [9,34,35,40,42,61]. Among these, myocarditis carries the highest incidence and fatality rate, with the worst prognosis [9,41,47,58]. In the multicentre study by Mahmood et al., 46% of patients with ICI-induced myocarditis developed MACE, with CV death accounting for 37.5% of all MACE cases [9]. The pharmacovigilance study by Salem et al. revealed that CV-irAEs were serious in the majority of cases (>80%), with fatalities occurring in 50% of myocarditis cases, 21.1% of pericardial disorders, and 6.1% of vasculitis (p<0.0001) [10].

Among 2478 individual case safety reports retrieved from the EudraVgilance database, 99.4% of reported CV-irAEs were classified as serious, with 25% having a favourable outcome and 30% resulting in a fatal outcome [67]. However, lethal suspected adverse events retrieved from spontaneous reporting systems must be interpreted with caution regarding their causal relationship with ICI therapy. Conversely, data derived from meta-analyses of clinical trials indicate that CV fatality rates vary between 0.32% and 3.2% [13-15,68-70]. Interestingly, some meta-analyses report no statistically significant differences in mortality due to CV-irAEs when comparing ICIs to alternative therapies [71-72].

In our review, the total CV fatality rate was observed to be 1% (95%CI: 0%, 3%) among study participants, placing it in the center of the range of estimates obtained from meta-analyses. This observation may be attributed to the prevalence of cardiac arrhythmias compared to myocarditis within this meta-analysis, with the latter being associated with the greatest mortality rate and the most adverse prognosis [10,32,47,67].

Time to First Cardiac Event and Risk Factors

The examination of short-term and long-term adverse events of these novel therapies is essential, given their indicated duration of treatment. The subsequent burden of CV risk factors is of great concern in patients with advanced cancers or cancer survivors, as they represent a leading cause of mortality [73]. Early CV-irAEs were initially described mainly as acute and fulminant myocarditis, often accompanied by hemodynamic failure, with a risk of death of up to 50% [8-10].

The time to onset (TTO) of CV-irAEs is usually very short, even after the first dose of ICI therapy, with the median time to occurrence being approximately 30 days [9,10,45,54,74,75]. The first 90 days following the initiation of ICI treatment are identified as a higher-risk period, highlighting the need for cardiac monitoring during this timeframe, especially for patients with risk factors [9,43,76]. Early-onset CV toxicities are mainly represented by acute, fulminant myocarditis, Takotsubo presentation [9,10,45,77], or pericardial disorders and vasculitis, likely associated with the inflammatory process [10].

Cases of late CV-irAEs (≥90 days after ICI therapy initiation) have been documented in cohort studies with clinical presentations different from those of early onset, showing an increased incidence of cardiac failure and left ventricular systolic dysfunction [28,32,36,78]. In the study of Dolladille et al., a comparison analysis between the early and late cardiac adverse events cases, in both the cohort and the Vigibase, showed that supraventricular arrhythmias and myocarditis were not usually observed in the late cases, and conversely, late cases exhibited more heart failure, left ventricular systolic dysfunction and pericardial disorders [78]. In our meta-analysis, the overall median time to CV-irAEs was determined to be 119 days (IQR 53-180), with a higher combined incidence of arrhythmias, cardiac failure, and ischemic heart disease compared to myocarditis and pericardial disease, confirming literature findings.

The occurrence of cardiotoxicity shortly after exposure to an ICI suggests a possible predisposition to such cardiotoxicity, which may be linked to pre-existing CV factors [32,33, 48,79] or an increased burden of comorbidities [36]. A large electronic medical records-based study from multiple healthcare organizations [28] showed that older age, male gender, black race, and a history of cardiac failure and MI were associated with an increased risk of developing new CV-irAEs, similar to the general population [80]. In the multicentre retrospective study by Mahmood et al., myocarditis was more frequent in patients with CV risk factors (diabetes, higher BMI) and those receiving dual ICI therapy [9]. The majority of pharmacovigilance studies report that dual ICI regimens significantly increased not only the risk for CV-irAEs but also fatality rates [66,76]. Furthermore, findings from retrospective studies indicate that older individuals, male patients, and those with lung cancer or diabetes exhibit an increased risk of experiencing MACE [34].

Another important risk factor for new CV events is the presence of prior irAEs [15,32,33,46]. Previous research has shown that around 50% of patients experiencing ICI-mediated myocarditis also present with concurrent irAEs, including conditions such as myositis and myasthenia gravis [9,81,82]. This association might reflect a shared antigen profile and immune phenotype between cardiac and skeletal muscle [81,82]. In our meta-analysis, the most frequently observed comorbidities in the CV-irAE population were hypertension (36%) and diabetes (14%), with a smaller percentage (6%) presenting with dyslipidemia and a history of ischemic heart disease. Additionally, lung cancer emerged as the predominant cancer type, accounting for 59% of cases, followed by melanoma at 11%, which aligns with the primary indications for these drugs.

Myocarditis

The pathophysiological mechanism of ICI-related myocarditis is not fully elucidated. Most theories have largely focused on a common or homologous antigen between the cardiomyocyte and tumor, mistakenly recognized as a foreign antigen, by CD4+ and CD8+ T-cells and CD68+ macrophages [8,9]. Many studies have shown that PD-1 and PD-L1 are constitutively expressed in mouse and human cardiomyocytes [83] and that CTLA-4 and PD-1 deletions are associated with autoimmune myocarditis in mice [84,85]. Also, lymphocytic infiltration observed in myocardial biopsies suggests that immune inflammation is a main pathogenesis [86,87]. Blockade of PD-1 by ICIs enhanced cardiomyocyte inflammation and apoptosis by enhancing the T-cell response [88,89]. Autoantibodies such as troponin antibody, myosin antibody, and β-adrenergic antibodies may also play a role in the pathogenesis of ICI-related myocarditis [90].

Myocarditis is the primary focus of current immunotherapy studies with a range of presentations: (i) subclinical probable myocarditis (absence of cardiac symptoms) accompanied by abnormal ECG, echocardiogram, or troponin results indicative of myocarditis, (ii) subclinical possible myocarditis (absence of cardiac symptoms) lacking abnormal ECG, echocardiogram, or troponin results, with only abnormal CV MRI findings, (iii) smoldering myocarditis an inflammation of the heart muscle, that is characterized by a lack of prominent symptoms or a presentation of mild, nonspecific symptoms, often accompanied by elevated cardiac biomarkers, and (iv) acute myocarditis, a condition where myocardium becomes inflamed, manifesting in various ways, from mild symptoms like chest pain to severe heart failure, arrhythmias, or even sudden cardiac death [8,9,41,47,58,75,91-93]. In the context of ICI therapy, the spectrum of myocarditis is presented from subclinical and smoldering early stages to acute myocarditis with less severe progression [94-96], to fulminant cases with high fatality rates [7,8,92]. It was initially described in several clinical studies as case reports [82,97], and likely underestimated in earlier research [98].

In meta-analyses of clinical trials, the incidence of grade 1-5 myocarditis fluctuated between 0.12% [68] among patients with various malignancies and 0.76% in patients with lung cancer [14]. A broader spectrum of occurrences has been noted in pharmacovigilance studies, with incidence rates differing from 0.23% [58] to 22% [67]. Variability is also apparent in real-world retrospective studies, where ICI-related myocarditis has been reported to range from 0.02% [48] to 6.97% [31]. On the other hand, fatality rates in pharmacovigilance studies and EMR records were near 50% [29,92,93] among patients who developed myocarditis, with a few exceptions where systematic screening led to earlier detection [41]. Timely identification is essential is critical, particularly when myocarditis presents with forms of arrhythmias, such as malignant ventricular arrhythmias and severe conduction block [75].

Our meta-analysis indicated that the pooled incidence of myocarditis was 11% (95%CI: 5%, 18%), making it the second most significant among all CV-irAEs. This rate exceeds the estimates derived from clinical trial meta-analyses [14,15,68,69,99] and most pharmacovigilance studies [10,100,101]. The variation in reported prevalences of myocarditis may be attributed to several factors. One explanation could be the possible occurrence of asymptomatic (subclinical) immune-related myocarditis, with certain events remaining unidentified or unreported [94-96]. Additionally, heterogeneity in clinical manifestation and reporting bias by investigators may contribute to the complexities associated with diagnosis [4,9-10]. This is the reason why various criteria like B-type natriuretic peptide (BNP) elevation, cardiac troponin T conversion, new-onset morphological ECG abnormalities, or biopsy-proven acute myocarditis must be integrated to ensure prompt diagnosis [33] 

Pericardial Disease

Among cancer patients, pericardial diseases often arise due to malignancy but may also develop secondary to treatment with traditional cytotoxic chemotherapy or radiation therapy [102,103]. Initial findings from RCTs have begun to indicate the occurrence of ICI-associated pericardial disease, especially in lung cancer patients [104]. Also, in case reports [105,106], pericardial involvement was predominantly reported in patients with lung cancer treated with ICIs, with a substantial mortality rate [105]. Meta-analyses of clinical trials have indicated that the incidence of pericardial disease in lung cancer populations ranges from 0.011% to 0.63% [12,14,107], while in multi-cancer populations, where lung cancer constituted nearly 50%, the incidence ranges from 0.08% to 0.5% [15,68,99,108].

The most extensive analysis of the World Health Organization’s (WHO) database indicated that pericardial disease accounted for 0.3% of all reported toxicities associated with ICIs, with the majority (56%) referring to lung cancer patients receiving anti-PD-1 or anti-PD-L1 therapy after radiotherapy [10]. Other pharmacovigilance databases reported that pericardial effusion and pericardial disease had incidences of 3.17% and 3.56%, respectively, among all CV-irAEs [65,74]. While these observations come from large population sample sizes, the real incidence of immune-related pericarditis in patients with advanced non-small cell lung cancer (NSCLC) in daily clinical practice remains unknown [53,56].

Our meta-analysis revealed that the overall incidence of pericardial disease was 5% (95%CI: 1%, 10%), positioning it as the fifth most prominent among all CV-irAEs, surpassing the results obtained from meta-analyses of clinical trial [12,14,15,68,99,107,108] and pharmacovigilance studies [65,74]. The most probable explanation is that the majority of our population comprises lung cancer patients (59%; 95%CI: 47%, 70%) (Table 2). Conversely, the lack of a control group or descriptive demographics to adjust for confounders [49] poses limitations in distinguishing whether pericardial (and pleural) diseases are common cancer complications or a result of the synergy between radiotherapy and immunotherapy [10,56].

Arrhythmias

The mechanisms underlying ICI-associated cardiac arrhythmias remain unclear but may involve inflammation of the His-Purkinje conduction system, myocarditis with inflammation and fibrosis, and other causes of arrhythmias in cancer patients (e.g., QT interval-prolonging drugs, electrolyte imbalances, myocardial metastases) [109]. Mere ICI-associated cardiac arrhythmias in the form of ventricular arrhythmias and conduction block might be a result of the T-lymphocyte-mediated inflammatory infiltration into the myocardium [10]

Due to an insufficient understanding of the forms of arrhythmias associated with ICIs, some case reports [110,111] and observational studies [75,79,91] have primarily linked arrhythmic events with myocarditis. Additionally, immune-mediated arrhythmias were disproportionately more frequently reported as concurrent cardiotoxicity [8,10,74,99], mainly cardiac failure, cardiac ischemia, pericardial disorders, and cardiac valve disorders, leaving the hypothesis of secondary to concurrent irAE complications rather than ICI treatment itself [10].

Meta-analyses of clinical trials suggest that the incidence of total arrhythmic events may vary from 0.014% to 2.48% when dual ICI therapy is employed [12,15,68,108]. From the EudraiVgilance database, signs and symptoms of arrhythmic cardiac events were mostly reported as AF and conduction disorders (AF 8.9%, tachycardia 5.5%, arrhythmia 3.2%, ventricular tachycardia 0.2%, bradycardia 0.08%, atrioventricular complete block 1.9%, cardiogenic shock 1.5%) [67]. From real-world observational studies, the total incidence of arrhythmias has been reported to vary between 0.98% and 11.64% [21,28,32,44,45,48,53]. In some of them, arrhythmias were identified as the most prevalent CV-irAEs, surpassing myocarditis, with AF being the most commonly observed type [29,44]. In the FDA Adverse Event Reporting System database, AF accounts for 4.84% of the cases, ranking among the top five cardiac adverse events documented in the database [65]. Subsequent pharmacovigilance studies aligned with this observation, revealing AF incidence rates between 4.8% [65] and 8.9% [67]. Also, ICI-associated cardiac arrhythmias may manifest as varying degrees of heart block, bradycardia, or even sudden cardiac death due to total heart block, which is associated with increased CV mortality [45,47,79,112,113]. Meta-analyses of clinical trials suggest that the incidence of cardiac arrest may vary from 0.06% to 1% [14,68,99,108,114].

The findings of our meta-analysis, encompassing all types of conduction disorders classified as arrhythmias, suggest an overall incidence rate of 18% (95%CI: 10%, 27%), without differentiating whether arrhythmias were independent events or secondary to myocarditis or other concurrent irAEs. This positions arrhythmic events as the most significant CV-irAEs, surpassing the results obtained from meta-analyses of clinical trials and pharmacovigilance studies. The most probable explanation for this discrepancy is the increasing surveillance for these events in healthcare facilities, which now categorize them as a separate entity among CV-irAEs following the widespread implementation of ICIs in cancer treatment.

Cardiac Failure

The primary evidence for immune-related cardiac failure primarily comes from case reports [115,116], due to the rarity of this condition in clinical trials and subsequent meta-analyses, which report an incidence rate ranging between 0.11% and 2.5% [14,68,99,108,114]. Results from pharmacovigilance studies show that cardiac failure ranks among the top five CV-irAEs, with reported incidences of 4% [65], 9.5% [67], and 12.8% [76]. In contrast, observational studies involving cancer patiens receiving immunotherapy reveal significant variations in the incidence of cardiac failure, ranging from 1.5% to 19% [29,32,33,45,46, 55].

These estimates are difficult to compare directly, suggesting that the risk of cardiac failure may be higher in certain patient groups compared to those seen in clinical trials or pharmacovigilance studies. In a nationwide Danish study, the relative rate of cardiac failure was only found to be increased in patients with lung cancer and PD1 inhibitors after six months, with a one-year risk of 2.5% [55]. Pre-existing CV factors, such as hypertension, diabetes, or dyslipidemia, in patients without a history of cardiac failure or echocardiographic abnormalities prior to ICI therapy, could predispose to subsequent cardiac failure events during ICI treatment [21]. When a history of cardiac failure is present, there should be a high index of clinical suspicion for CV-irAEs, as there is an increased risk of acute decompensated heart failure or worsening of pre-existing heart failure [33]. In some retrospective observational studies [21,45], heart failure was more common than myocarditis. In clinical practice, late manifestations of immune-related myocarditis may present as heart failure symptoms, suggesting a complex relationship between various ICI-related cardiotoxicities [78,117].

In our meta-analysis, cardiac failure ranked third in terms of incidence, recorded at 8% (95%CI: 2%, 15%). This finding exceeds the incidence rates reported in clinical trials and pharmacovigilance studies, perhaps due to the high prevalence of pre-existing CV factors, such as hypertension, diabetes, or dyslipidemia, and history of cardiac failure and ischemic heart disease (Table 2). These comorbidities highlight the importance of close monitoring for diagnosing this CV-irAE in certain patient groups.

Takotsubo Cardiomyopathy

Early cases of Takotsubo cardiomyopathy associated with ipilimumab treatment have been reported in patients with melanoma [118]. Subsequently, a limited number of cases of Takotsubo-like syndrome (TTS) have emerged as a new manifestation of ICI-related cardiotoxicity [119,120]. However, the relationship between TTS and immunotherapy remains unclear due to its rarity and the concurrent occurrence of other cardiotoxicities, such as myocarditis and pericarditis [121,122]. A history of malignancy appears to be an independent risk factor for TTS, possibly due to the intense physical and emotional stress experienced by cancer patients [123].

According to the EudraVigilance database [67], cardiomyopathy had an incidence of 2.5%. Potential stressors identified included the presence of non-cardiac irAEs and other risk factors, such as active smoking and cardiovascular comorbidities, may explain this result [122]. In the descriptive observational analysis by Escudier et al., TTS appeared in a high proportion, reaching 14%, because of the lack of a referral population [79].

In our meta-analysis, the number of Takotsubo cardiomyopathy cases was notably low, with only 65 cases identified out of 54,409. The pooled incidence rate was calculated at <0.2% which may indicate a misdiagnosis of TTS, potentially confused with myocarditis or MI. This outcome emphasizes the importance of utilizing appropriate diagnostic criteria and recognizing the specific group of cancer patients who are more prone to encounter this rare CV-irAE.

Coronary Artery Disease and Vascular Events

Myocardial ischemia and MI are life-threatening CV-irAEs that have been less frequently reported in the literature [124-126]. The development of ACS induced by ICIs was initially postulated [109], as immune checkpoint proteins play critical roles as negative regulators of atherosclerosis [127]. Both preclinical and clinical evidence suggest that ICIs may accelerate atherosclerosis or increase atherosclerotic coronary plaques' destabilization and rupture [128,129]. A meta-analysis of RCTs estimated that ICI users had more than a threefold increased risk of myocarditis and dyslipidemia, and twice the risk of MI and ischemic stroke compared to non-ICI users [130]. Estimates regarding the incidence of MI, obtained from meta-analyses of clinical trials, suggest a range between 0.02% and 2.56% [14,15,68,99,108,114]. A broader spectrum of occurrences has been noted in pharmacovigilance studies. According to the EudraVigilance database [67], the incidence of acute MI was 2.66%, while in the WHO’s VigiBase, as of November 2019, MI ranked among the top four CV-irAEs, with an incidence of 17.6% [76].

In observational studies, the use of ICIs was associated with a higher risk of atherosclerotic CV events, such as MI and coronary revascularization [32,45,50,59], although some cohorts of NSCLC patients [42,61] reported no association. Subgroup analyses showed that atherosclerotic CV events were more common in men [46], in patients with a history of cardiovascular disease [38], or those with concurrent irAEs [46]. These events typically occurred within the first six months of ICI initiation [38,46,62], suggesting a short-term increase in CV risk due to plaque destabilization.

In our meta-analysis, ischemic heart disease ranked fourth in terms of incidence, with an overall rate of 6% (95%CI: 3%, 11%) exceeding the incidence rates reported in meta-analyses of clinical trials [14,15,68,99,108,114] and some pharmacovigilance studies [67]. The term ischemic heart disease includes ACS, MI, NSTEMI, STEMI, and unstable angina. A challenging aspect of interpreting these results lies in the diagnostic complexities of ACS, which may also involve vasculitis or focal myocarditis [109]. Especially, immune-associated myocarditis is mentioned with a wide spectrum of disease severity, including pseudo-ACS [131] or type 2 MI secondary to other causes within the period of immunotherapy [132]. Timely and precise diagnosis holds significant importance because treatment modalities of ACS and immune-related myocarditis differ considerably, with the latter primarily depending on the immediate initiation of immunosuppressive therapy [91].

Regarding vascular events, both arterial thromboembolic events (ATE) and venous thromboembolic events (VTE) have been linked to ICI treatment in case series [133] and cohort studies [40,50,51]. Otherwise, every 10th patient experiences VTE during ICI therapy, and every 50th patient suffers an ATE [51]. In particular, patients treated with ICIs had an increased risk of arterial thrombotic events, specifically ischemic stroke (HR, 3.0; 95%CI: 1.0-9.0) and pulmonary embolism (HR, 5.5; 95%CI: 1.4-21.3) [40]. Regarding VTE, estimates from retrospective cohort studies indicate an incidence of 9.9% [52] and 13.8% [60]. Higher rates of VTE were observed in patients with combination ICI compared with ICI monotherapy and with conventional chemotherapy (six-month cumulative incidence rate of 9.3% vs 2.6% and 4.67%) [50]. Most VTEs occurred within six months of ICI initiation, while patients are on active ICI treatment [52]. Mechanistically, for VTE, ICIs could induce a systemic proinflammatory status, which in turn might enhance the prothrombotic state by activation of coagulation and platelets and impairment of fibrinolysis [127].

In our meta-analysis, arterial disease shared the fifth place (5%; 95%CI: 1%, 12%) and was higher regarding VTE, which ranked sixth (3%; 95%CI: 0%, 8%). This could be elucidated by the fact that within the term ATE, we have encompassed a range of clinical conditions like stroke, transient ischemic attack, vasculitis, pulmonary artery hypertension, acute limb ischemia, and calcified plaque at the aortic arch. Although numerous reports have linked ATE and VTE to ICI treatment, further research is needed to clarify this association. Particularly, the condition of cancer is an independent risk factor for VTE, and this risk is significantly elevated compared to individuals without cancer [134,135]

Limitations

The present systematic review has several limitations that should be considered. The studies included in this analysis were observational in nature, with some data sourced from healthcare electronic databases [28,42,43,46,48], which introduces a potential lack of event adjudication and selection biases. We have omitted observational studies conducted in non-English languages, as well as those where the primary endpoint was not a CV-irAE, which may have resulted in missing data. Most of the studies employed a retrospective design due to the lack of a standardized prospective CV screening protocol across the various sites. The decision to screen for cardiac biomarkers and conduct additional tests was left to the discretion of the healthcare providers, which may have affected the promptness and accuracy of diagnoses. Many studies were also characterized by limited sample sizes, which may lead to potential confounding factors and reporting bias. Direct comparisons between clinical trials, pharmacovigilance studies, and cohort studies present certain limitations due to inherent design differences (e.g., prospective vs. retrospective, passive vs. active reporting). To the best of our knowledge, this represents the first meta-analysis of observational studies focusing specifically on CV-irAEs. As such, these findings are of significant importance, highlighting the need for further comprehensive research in this field, particularly in the development of consensus diagnostic criteria to improve case ascertainment and reporting.

## Conclusions

We reviewed the incidence of CV toxicities in cancer patients treated with ICIs in observational studies, which was incompletely characterized. The prevalent CV-irAEs included arrhythmias, myocarditis, and cardiac failure, often resulting in life-threatening complications. Post-market surveillance studies provide higher estimates and valuable insights into the long-term prevalence and outcomes of these patients than meta-analyses of clinical trials and pharmacovigilance studies. Prospective cohort studies with standardized CV monitoring and development of risk stratification tools would be more appropriate in order to investigate the association between ICIs and CV toxicity, especially for high-risk patients, such as elderly patients and those with pre-existing CV conditions.
